# Exercise Prevents Diaphragm Wasting Induced by Cigarette Smoke through Modulation of Antioxidant Genes and Metalloproteinases

**DOI:** 10.1155/2018/5909053

**Published:** 2018-03-29

**Authors:** Gracielle Vieira Ramos, Alessandra Choqueta de Toledo-Arruda, Clara Maria Pinheiro-Dardis, Camila Liyoko Suehiro, Thiago Luiz de Russo, Rodolfo Paula Vieira, Milton Arruda Martins, Tania Fátima Salvini, João Luiz Quagliotti Durigan

**Affiliations:** ^1^Physical Therapy Division, University of Brasília and Department of Physical Therapy, Universidade Paulista, Brasília, DF, Brazil; ^2^Department of Medicine (LIM 20), School of Medicine, University of Sao Paulo, Sao Paulo, SP, Brazil; ^3^Postgraduate Program in Sciences of Human Movement and Rehabilitation, Federal University of São Paulo (UNIFESP), Av. Ana Costa 95, 11060-001 Santos, SP, Brazil; ^4^Department of Physical Therapy, Federal University of São Carlos, São Carlos, SP, Brazil; ^5^Postgraduate Program in Bioengineering, Universidade Brasil, Rua Carolina Fonseca 235, Itaquera, 08230-030 São Paulo, SP, Brazil; ^6^Brazilian Institute of Teaching and Research in Pulmonary and Exercise Immunology (IBEPIPE), Rua Pedro Ernesto 240, 12245-520 São José dos Campos, SP, Brazil; ^7^Physical Therapy Division, University of Brasília, Brasília, DF, Brazil

## Abstract

**Background:**

The present study aimed to analyze the effects of physical training on an antioxidant canonical pathway and metalloproteinases activity in diaphragm muscle in a model of cigarette smoke-induced chronic obstructive pulmonary disease (COPD).

**Methods:**

Male mice were randomized into control, smoke, exercise, and exercise + smoke groups, which were maintained in trial period of 24 weeks. Gene expression of kelch-like ECH-associated protein 1; nuclear factor erythroid-2 like 2; and heme-oxygenase1 by polymerase chain reaction was performed. Metalloproteinases 2 and 9 activities were analyzed by zymography. Exercise capacity was evaluated by treadmill exercise test before and after the protocol.

**Results:**

Aerobic training inhibited diaphragm muscle wasting induced by cigarette smoke exposure. This inhibition was associated with improved aerobic capacity in those animals that were submitted to 24 weeks of aerobic training, when compared to the control and smoke groups, which were not submitted to training. The aerobic training also downregulated the increase of matrix metalloproteinases (MMP-2 and MMP-9) and upregulated antioxidant genes, such as nuclear factor erythroid-2 like 2 (NRF2) and heme-oxygenase1 (HMOX1), in exercise + smoke group compared to smoke group.

**Conclusions:**

Treadmill aerobic training protects diaphragm muscle wasting induced by cigarette smoke exposure involving upregulation of antioxidant genes and downregulation of matrix metalloproteinases.

## 1. Introduction

Chronic obstructive pulmonary disease (COPD) is associated with respiratory and peripheral muscle dysfunction. REDOX imbalance induced by cigarette smoke (CS) exposure damages lipids, proteins, and nucleic acids and is involved in diaphragm remodeling and muscle atrophy in COPD [1, 2]. However, little is known regarding the key role of extracellular matrix (ECM) remodeling associated with oxidative stress on respiratory muscles weakness in COPD during chronic CS exposure. Metalloproteinases (MMPs), MMP-2, and MMP-9 are proteolytic enzymes that play an essential role in ECM remodeling of skeletal muscle [3–6]. Studies have showed that MMPs also are involved in inflammation and development of interstitial fibrosis of many organs [7–9]. In addition, Yao et al. 2013 [10] have showed in mice and humans a contribution of the imbalance between tissue inhibitors of MMPs (TIMPs) and MMPs, notably between TIMP-1 and MMP-9 [10]. Increased gene expression and activity of MMP-9 and MMP-2 in diaphragm muscle were also observed, which can contribute to the skeletal muscle myopathy during heart failure [11, 12].

It is well described that REDOX imbalance has a positive correlation with upregulation of MMPs [13, 14]. Rajagopalan et al. (l996) [14] noticed that increased levels of free radicals, both reactive oxygen species (ROS) and reactive nitrogen species (RNS), induced activation of MMP-2 and MMP-9 in cultured smooth muscle cells. Kameda et al. (2003) [13] also showed increased activity of MMP-2 and MMP-9 and oxidative stress, resulting in ventricular remodeling (enlargement), followed by increased rate of morbidity and mortality of patients with coronary artery disease. Although the regulation of these enzymes is associated with oxidative stress in heart and lungs, their expression in the diaphragm after chronic CS exposure remains unknown.

Nuclear transcriptional factor (erythroid-derived 2)-like 2 (NRF2) pathway constitutes a regulatory pathway against nocive effects caused by oxidative stress through antioxidant genes upregulation [15, 16]. Some studies have suggested that, in physiological conditions, NRF2 is maintained in the cytosol by a cluster of proteins, including its cytosolic inhibitor, kelch-like ECH-associated protein 1 (KEAP1). However, during stressful responses, redox imbalances, and cancer evolution, it dissociates from KEAP1 and translocates to the nucleus to regulate the transcription of antioxidant genes [17], such as heme-oxygenase1 (HMOX1). This molecular mechanism has been observed in liver, lungs, and kidneys through KEAP1 knockdown mice approach. However in the skeletal muscle this pathway remains unclear [18]. Furthermore, a recent study has showed that NRF2 also could be associated with regulation of MMPs. In this report higher levels of MMP-9 activity in (−/−) NRF2 knockdown mice were noticed when compared to NRF2 (+/+) mice after Spinal Cord Injury (SCI), suggesting that NRF2 plays a protective role in spinal cord, possibly by limiting the inflammatory response that occur after SCI via MMP-9 modulation [19]. Despite the fact that some studies have reported the critical role of NRF2 in the modulation of inflammation [19, 20], little is known about the crosstalk between MMPs and NRF2 in ECM remodeling, especially in diaphragm muscle exposed to CS [21, 22]. Aerobic training in COPD patients alleviates symptoms, improves quality of life, and decreases exacerbation episodes and the risk of mortality in COPD. Moreover, there are evidences that aerobic training has an essential role in the prevention and progression of pulmonary disease due to cigarette smoking [23]. Interestingly, some reports showed that aerobic training also decreases the incidence of a wide range of ROS-associated diseases, including heart disease, type II diabetes, cigarette smoke-induced pulmonary disease, and certain types of cancers through the improvement of antioxidant defenses [23, 24]. Although the literature presents a consensus about the essential role of NRF2 pathway for the protection against oxidative stress, there is no evidence about activity of NRF2 and HMOX1 pathways after aerobic training in mice exposed to cigarette smoke. In addition, there is limited information on how physical training can affect ECM turnover in respiratory muscles as well as oxidative and antioxidative response to protect skeletal muscle against CS. Therefore, the goal of this study was to analyze the effects of physical training in the NRF2-HMOX1, an antioxidant canonical pathway, and the MMP-2 and MMP-9 activities in diaphragm muscle after CS exposure. We hypothesized that chronic CS exposure would increase the MMPs activity and decrease the expression of antioxidant enzymes in diaphragm muscle, while aerobic conditioning would result in an opposite and positive effect.

## 2. Material and Methods

### 2.1. Animals and Experimental Design

Male C57BL/6 mice (6–8 weeks; 20.10 ± 4.73 g) were randomized into four groups of 8 animals per group: (1) control; (2) smoke (exposed to cigarette smoke); (3) exercise (submitted to treadmill training); and (4) exercise + smoke (submitted to treadmill training and exposed to cigarette smoke). The animals were housed in plastic cages under controlled environmental conditions (12-hour light/dark cycle) with free access to water and standard chow (Socil, São Paulo). The protocol of the present study was approved by the ethical committee of Faculdade de Medicina da Univesidade de São Paulo (São Paulo-Brazil) and was developed in compliance with the “Guide for care and use of laboratory animals” [25].

### 2.2. Cigarette Smoke Exposure Protocol

In order to induces COPD, animals were subjected to 12 commercially filtered cigarettes per day (0,8 mg of nicotine, 10 mg of tar, and 10 mg of CO per cigarette), corresponding to a total particulate matter concentration of 354.8 ± 50.3 mg·m^−3^·day^−1^. The CS exposure was performed placing the animals into a box (inhalation chamber) maintaining controlled CO levels (250 to 350 ppm) for 30 min a day^−1^, 5 days a week^−1^ for 24 weeks. Control animals were exposed to the same protocol, but using room air [23]. In addition, carboxyhemoglobin concentration in smoke-exposed animals was kept at 10% (±1,3%).

### 2.3. Treadmill Aerobic Training and Test

After an adaptation to aerobic treadmill training (3 days, 15 min a day^−1^, 25% inclination and 0.2 km/h^−1^), a physical test was performed to evaluate the maximal exercise capacity (100%), as previously described [23]. Animals were trained at 50% of maximal exercise capacity for 60 min/day, 5 days per week for 24 weeks, and CS exposure was always performed after 1 hour of physical training. Aerobic training started on the same day as the beginning of cigarette smoke exposure and continued until the end of the experimental protocol [23].

### 2.4. Zymography

Diaphragm muscle was chosen because it is the most important respiratory muscle during quiet and effort breathings. Mice were weighed and diaphragm muscles were carefully removed, weighed, and frozen for zymography evaluation. Tissue extraction and zymographic analysis were performed according to current methodology [26]. Briefly, equal amounts of total protein (30 *μ*g/lane), consisting of a pool of five animals per group (6 *μ*g per animal), were subjected to electrophoresis in triplicate. Zymography gels consisted of 10% polyacrylamide impregnated with gelatin at a final concentration of 100 mg/ml H_2_O in the presence of sodium dodecyl sulfate (SDS), under nonreducing conditions. After 2 hours of electrophoresis (100 V), the gels were washed twice for 20 minutes in a 2.5% Triton X-100 solution and incubated at 37°C for 20 h in a substrate buffer (50 mM Tris-HCl, pH 8.5, 5 mM CaCl_2_, and 0.02% NaN_3_). The gels were stained with Coomassie Brilliant Blue R-250 for 30 minutes and stained in methanol and acetic acid for 20 minutes. Gelatin-degrading enzymes were visualized as clear white bands against a blue background, indicating proteolysis of the substrate protein. The samples were also assayed in presence of 15 mM EDTA that inhibited MMP activity. The molecular mass of gelatinolytic activities was determined by comparison to reference protein molecular mass marker, PageRulerTM Prestained Protein Ladder (Fermentas Life Sciences, Burlington, ON). Activity bands were identified following previously description, according to their molecular weights (92 kDa: pro-MMP-9; 81 kDa: active-MMP-9; 72 kDa: pro-MMP-2; 66 kDa: intermediary-MMP-2; 62 kDa: active-MMP-2) [26]. The bands found in all groups were 72–62 kDa, suggesting the activation of MMP-2 as proposed by [27]. Active bands were emphasized as described by [28]. Data is expressed as concentration of MMP-2 (i.e., the totality of integrated optical density for the MMP-2 proenzyme, intermediate, and active forms) and MMP-2 active form. Densitometry quantitative analysis of the protein bands in the zymography was performed using GeneTools v3.06 software (Syngene, Cambridge, UK).

### 2.5. Gene Expression by Real-Time PCR

Total RNA was isolated from diaphragm muscle using TRI-Reagent (Sigma, St. Louis, MO, USA). RNA quantity and quality were assessed using RNA Nano 6000 kit on a 2100 Bioanalyzer capillary electrophoresis system (Agilent, Santa Clara, CA, USA). Reverse transcription was carried out using High-Capacity cDNA Reverse Transcription Kit with RNase Inhibitor (Applied Biosystems). Gene expression was evaluated by quantitative real-time PCR. Two microliters of each reverse transcription product were amplified in 20 L of 1X reaction buffer (TaqMan® Universal PCR Master Mix, Applied Biosystems) using appropriate TaqMan gene expression assays in a Step One Plus Thermocycler (Applied Biosystems) for the following genes:* NRF2* (Mm00477784_m1);* KEAP1* (Mm00497268_m1); and* HMOX1 *(Mm00516005_m1). Abundance of mRNA for different genes in the diaphragm muscle was calculated with the use of 2^−ΔΔCt^ method and Beta-actin was used as the reference gene [29].

### 2.6. Statistical Analysis

Kolmogorov–Smirnov and Levene tests were used to analyze the normality and homogeneity of variance. Two-way ANOVA (factors: smoke and exercise) was used to identify differences among groups. When differences were observed, Tukey test was performed. Differences were considered significant when *p* < 0.05. All data were analyzed using the Statistica 7.0 software package (StatSoft Inc., Tulsa, OK, USA).

## 3. Results

### 3.1. Aerobic Exercise Capacity

**Figure 1:**
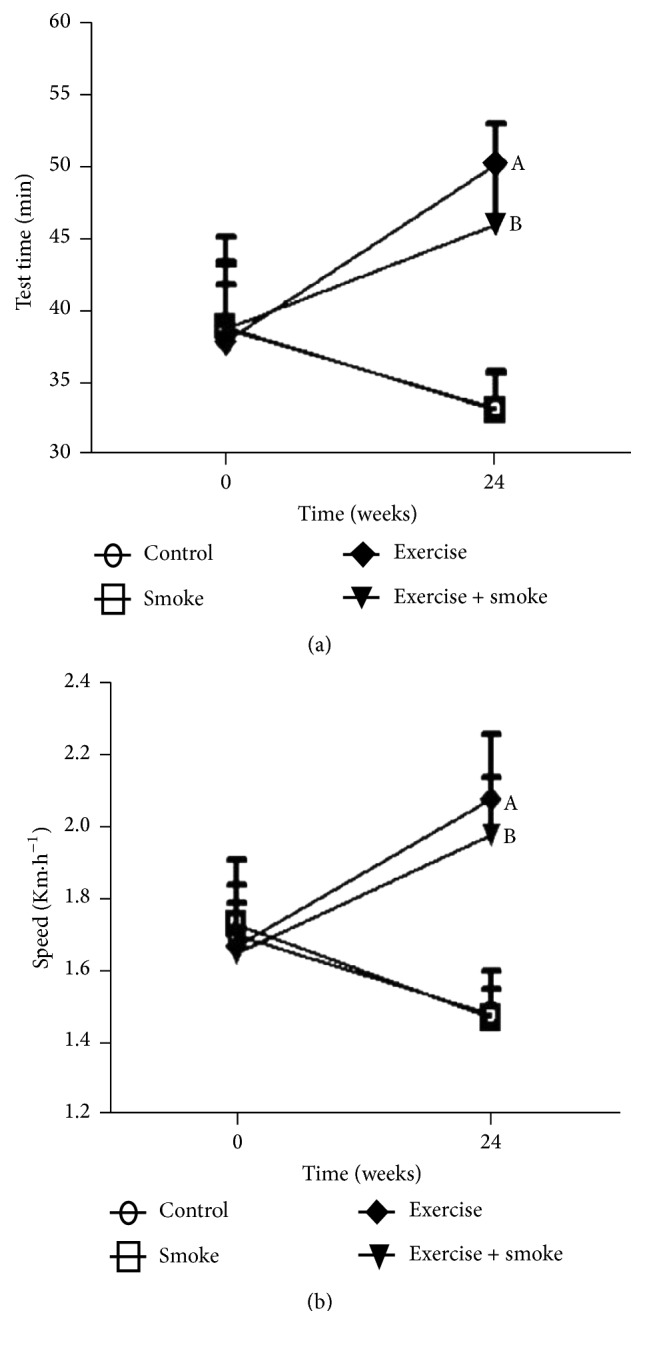
Results of the tests performed on the treadmill to evaluate the intensity of aerobic conditioning of the studied mice. Tests were performed before the beginning of the exposure to cigarette smoke (time 0) and 24 weeks of the protocol. (a) Time that the mice could exercise in the treadmill; (b) maximal speed reached by each mouse among the four experimental groups. Data are presented as means and standard deviation. A = *p* < 0.05: compared to control group. B = *p* < 0.05: compared to smoke group.

After 24 weeks of aerobic training, the exercise time on treadmill and the maximal speed were significantly greater in those groups that trained endurance exercise, when compared to the groups that were not trained. In Figure 1, aerobic capacity improvement is noticed in the exercise group, compared to control one (*p* < 0.05; Figure 1), as well as in exercise + smoke group compared to smoke group (*p* < 0.05; Figure 1).

### 3.2. Body and Muscle Weight

#### 3.2.1. Body Weight

Curiously, 24 weeks of cigarette smoke exposure caused an increase in body weight in both groups (smoke and smoke + exercise groups) that receive smoke tobacco when comparing their initial and final weights and when comparing them to the control group (*p* < 0.05). Moreover, body weight decrease was observed in control and exercise group, when comparing their initial and final weights (*p* < 0.05; Table 1).

#### 3.2.2. Diaphragm Muscle Weight

The chronic cigarette smoke exposure reduced significantly the weight of the diaphragm muscle compared to control (*p* < 0.05; Table 1). However, it was also noticed that aerobic exercise prevented muscle weight loss in exercise + smoke group, when compared to the smoke group that was not trained. In the control and exercise groups no changes were observed after 24 weeks of training.

### 3.3. Gene Expression by qPCR

#### 3.3.1. *NRF2*

**Figure 2:**
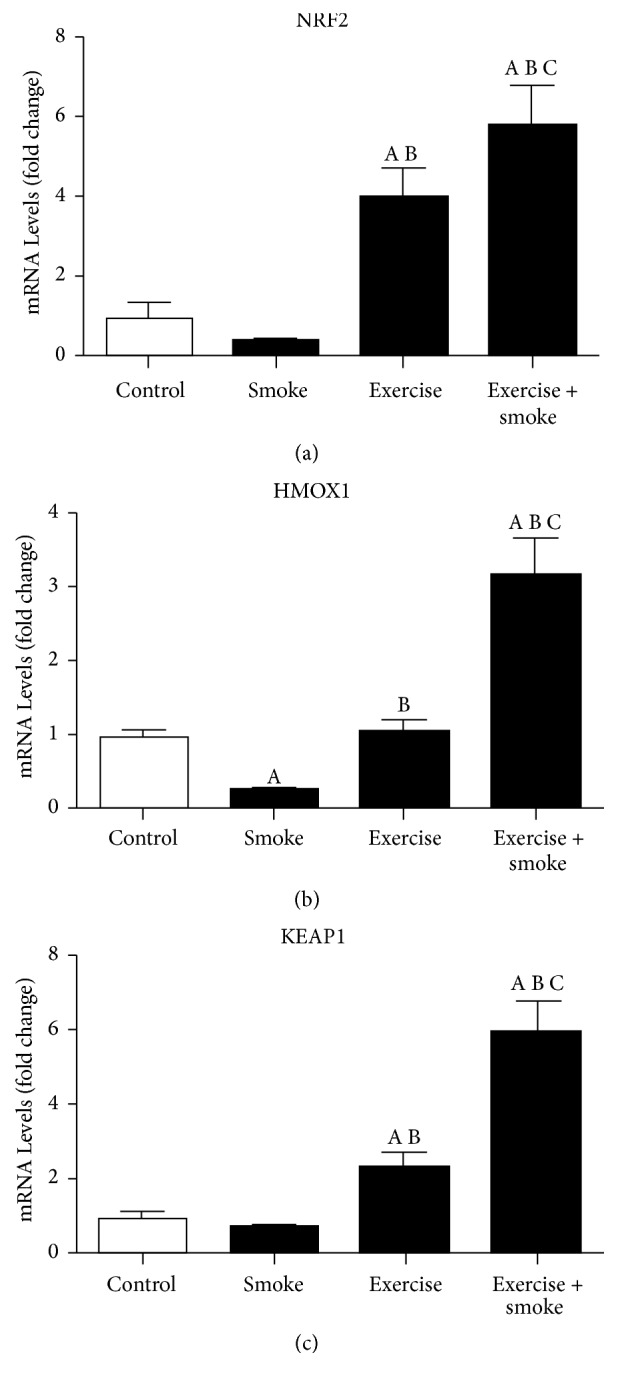
The messenger RNA (mRNA) levels of nuclear factor erythroid-2 like 2 (NRF2), heme-oxygenase1 (HMOX1), and kelch-like ECH-associated protein 1 (KEAP1) of diaphragm muscle. Data are expressed as mean ± standard deviation. A = *p* < 0.05: compared to control group; B = *p* < 0.05: compared to smoke group; C = *p* < 0.05: compared to exercise group.

Aerobic training, in exercise group, upregulated* NFR2* gene expression in approximately four times, when compared to control and smoke groups (*p* < 0.05, resp.). There was also* NRF2* gene expression increase in the exercise + smoke group, compared to the smoke and control group (*p* < 0.05, resp.). Different from the expected, no differences in* NRF2* gene expression between smoke and control groups were observed. However, exercise + smoke group presented increased* NRF2* gene expression, when compared to exercise group (*p* < 0.05, resp.) (Figure 2(a)).

#### 3.3.2. *HMOX*

In relation to* HMOX1* gene expression that is transcriptionally regulated by* NRF2, *no difference was found between exercise and control group. On the other hand,* HMOX1* gene expression decrease was found in smoke group, when compared to control (*p* < 0.05, resp.). Despite HMOX1 downregulation in smoke group, aerobic training promoted increases in* HMOX1* gene expression in exercise + smoke group, in comparison to control, smoke, and exercise groups, according to what could be seen in Figure 2(b). (*p* < 0.05, resp.)

#### 3.3.3. *KEAP1*

Similar regulation between NRF2 and KEAP1 was noticed in qPCR.* KEAP1 (NFR2 inhibitor)* gene expression increase was observed, in exercise group, when compared to control and smoke group (*p* < 0.05, resp.). Similar data was observed in exercise + smoke group, when compared to other groups (control, exercise, and exercise + smoke) (*p* < 0.05, resp.). An increase of* KEAP1* gene expression in the exercise + smoke group compared to exercise group is also observed (*p* < 0.05, resp.). These results suggest a possible counter-regulatory mechanism between* KEAP1* and* NRF2* (Figure 2(c)).

### 3.4. MMP-2 and MMP-9 Activity of Diaphragm Muscle

**Figure 3:**
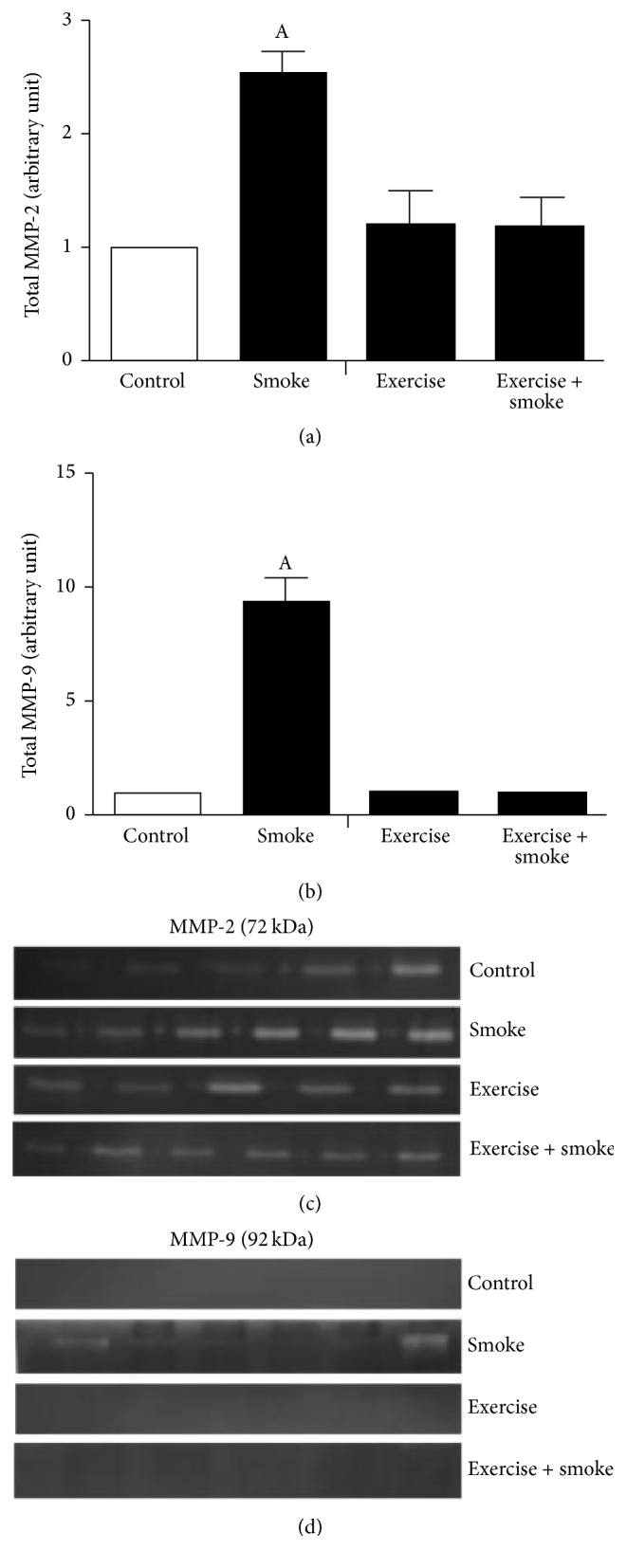
Representative gelatinolysis activity and gelatin zymographies in diafragham muscle of mice chronic cigarette smoke exposure. (a) Densitometry metalloproteinase-2 (MMP-2) gelatinolysis activity groups; (b) densitometry metalloproteinase-9 (MMP-9) gelatinolysis activity groups; (c) MMP-2 gelatin zymographies; (d) MMP-9 gelatin zymographies. A = *p* < 0.05: compared to control group.

The total MMP-2 activity and MMP-9 activity were elevated only in smoke group, compared to all experimental groups (control, exercise, and exercise + smoke) (*p* < 0.05; Figure 3(a)). No differences were found among exercise, exercise + smoke, and control groups (*p* < 0.05; Figure 3(b)).

### 3.5. Discussion

Our results indicate the importance of aerobic training over ECM remodeling through MMPs and oxidative stress in diaphragm muscle mass regulation. Aerobic training maintained MMP-2 and MMP-9 activities in basal levels and increased* NRF2* and* HMOX1* expression (antioxidant genes), which could be associated with protective effects of physical training during chronic CS exposure. These results are of particular interest since, in humans, COPD is the most common respiratory disease, with progressive and irreversible decline of lung function, in which the diaphragm dysfunction presents an important role. We showed that mice exposure to CS for 24 weeks decreased diaphragm muscle weight ~40% compared to the control group, suggesting that CS activated muscle proteolysis. We believe that muscle proteolysis observed in diaphragm muscle could be associated with inflammatory reaction and excessive ROS production. This hypothesis could be reinforced, since a decrease in* HMOX1* gene expression and MMP-9 (inflammatory marker) activity increase were observed in smoke group, compared to the control group. On the other hand, the results also showed that aerobic training is a potential intervention to prevent the imbalance between MMPs and antioxidant genes, caused by tobacco, in diaphragm muscle. Therefore, these results suggest that aerobic training has controlled muscle mass wasting through the regulation of antioxidant pathways.

Some studies have shown a regulatory mechanism between HMOX1 and transcriptional factor NRF2. Clinical and experimental studies have shown that NRF2 downregulation in the lungs of COPD patients and mice caused more susceptible development to emphysema after exposure to cigarette smoke [30–32]. Interestingly, in the present study, increase in* NRF2* gene expression was observed in the exercise + smoke group, compared to the smoke group. It is possible to suggest that aerobic training plays a key role mediating the upregulation of the antioxidant system, in an attempt to protect muscle cells against ROS, in special, in diaphragm muscle, which was analyzed here. Curiously, we did not demonstrate difference in* HMOX1* gene expression between control and exercise group. Therefore, other mechanisms might be involved in the regulation between NRF2 and HMOX1 in physiological conditions [30].

It is well described that KEAP1 is a regulatory bind protein of NRF2 in the cytosol facilitating its degradation by 26S proteasome [30, 31, 33, 34] Therefore, we might expect that* KEAP1* would be downregulated compared to* NRF2*, in both exercise and exercise + smoke. Different from what we expected, an increase of* KEAP1* in those groups was observed. The increase of* KEAP1* could be a cell mechanism to balance an excessive increase of* NRF2*, which is involved in the transcription of antioxidant target gene [30]. However, an increase of* KEAP1* gene expression between smoke and control group was not observed. We believe that the expression of KEAP1 at basal levels might be enough to inhibit NRF2 migration into nucleus and consequently inducing antioxidants transcription, but further studies are necessary to better understand the relation between KEAP1-NRF2 pathway in exercise and CS models.

The role of MMPs on ECM remodeling related to diaphragm muscle mass wasting has been poorly investigated. Our results support that CS exposure increases MMP-2 and MMP-9 activities, which might be associated with diaphragm mass muscle wasting in mice, whereas animals that were exposed to CS and submitted to exercise maintained MMPs activities similar to control group. Although the present study would not provide a direct regulatory mechanism between MMPs (MMP-2 and MMP-9) and antioxidant pathway (NRF2-HMOX1), this is the first report demonstrating the protective mechanisms induced by exercise on diaphragm muscle mass decrease following CS exposure. The data reported in the present study could be reinforced by study published by Mao et al. in 2010 [19] that proved that NRF2 has a critical role in MMP-9 upregulation of mice which were knockdown to NRF2. In addition, Hindi et al. 2013 [5] showed in dystrophic muscle of mdx mice that MMP-9 inhibition improves proliferation and engraftment of myogenic cells. According to this study, the role of MMP-9 in Duchene Muscular Dystrophy could be mediated by activation of proinflammatory cytokines leading to increased inflammatory response and degradation of ECM components [5, 35]. Future investigations will be necessary to prove the relation among NRF2, MMPs, and muscle remodeling.

It is well known that the activation of MMP-2 and MMP-9 in skeletal muscle occurs despite exercise intensity and varies according to the type of muscle evaluated [26, 36]. Durigan and colleagues (2009) [26] observed that treadmill training at low or moderate intensities (resp., 50% or 75% of maximal speed reached in the test) during 60 minutes a day, 5 days a week for 12 weeks, did not alter MMPs activity of diaphragm muscle. These data are in agreement with the data presented here, suggesting that a training protocol at moderate intensity (50% of maximal speed) maintained ECM remodeling in normal condition beneficiating the maintenance of diaphragm muscle remodeling in the exercise + smoke group.

In fact, training protocol at moderate intensity of exercise attenuated the harmful effects of CS exposure, regarding both MMP-9 and MMP-2 activities, in diaphragm muscle mass decrease. These results could be associated with the reduction of diaphragm muscle myopathy in CS, as previously described by Carvalho et al. (2006) [11] during heart failure. It was reported that mouse model of CS induces parenchyma mechanical dysfunction and lung remodeling, leading to impaired respiratory mechanic [23]. Probably, these mechanical changes could increase diaphragm muscle workload during training, since an increase has been observed in MMP-2 and MMP-9 activities only in CS animals. However, exercise training tends to soften the enhancement of MMPs activities, inherent to CS exposure. It is interesting to notice that, during physical exercise, abnormal dynamic in the ventilatory pattern can be observed in flow-limited patients, resulting in functional inspiratory muscle weakness by maximally shortening the muscle fibers in the diaphragm [37, 38]. The combination of excessive mechanical loading and increased velocity of shortening of the inspiratory muscles can also predispose them to fatigue [39]. As we did not observe diaphragm ECM remodeling in a mice model of COPD in trained group, it is possible to suggest that training did not lead to abnormal dynamic ventilatory and disadvantages of diaphragm, regarding force generation.

## 4. Conclusion

Aerobic training protects diaphragm muscle wasting induced by chronic CS exposure involving upregulation of antioxidant pathway,* NRF-2/HMOX*, and downregulation of the matrix metalloproteinases MMP-2 and MMP-9.

## Figures and Tables

**Table 1:** Final body weight and diaphragm muscle weight of experimental groups.

	Control	Smoke	Exercise	Exercise + smoke
Initial body (g)	38.79 ± 3.33	38.56 ± 5.56	39.51 ± 6.39	39.53 ± 4.14
Final body (g)	33.59 ± 2.59^§^	50.16 ± 3.09^a§^	33.34 ± 2.70^§^	46.44 ± 4.36^a§^
Gain (%)	13.41	30.08^§^	15.62	17.48^§^
Diaphragm weight (mg)	98.8 ± 23.2	60.1 ± 13.6^a^	89.5 ± 39.4	109.9 ± 19.6^b^

Normal diaphragm muscle (control); exercise in the treadmill (exercise); group exercise in the treadmill associated to chronic cigarette smoke exposure (exercise + smoke); group submitted to chronic cigarette smoke exposure (smoke). Data are expressed as mean ± standard deviation. a = *p* < 0.05: compared to control group; b = *p* < 0.05: compared to smoke group;  ^§^*p* < 0.05: intragroup comparison.
